# Case report for an internet- and mobile-based intervention for internet use disorder

**DOI:** 10.3389/fpsyt.2023.700520

**Published:** 2023-07-25

**Authors:** Karina Bernstein, Anna-Carlotta Zarski, Emilia Pekarek, Michael Patrick Schaub, Matthias Berking, Harald Baumeister, David Daniel Ebert

**Affiliations:** ^1^Clinical Psychology and Psychotherapy, Friedrich-Alexander-Universität Erlangen-Nürnberg, Erlangen, Germany; ^2^eHealth in Clinical Psychology, Philipps-University of Marburg, Marburg, Germany; ^3^Swiss Research Institute for Public Health and Addiction, Associated to the University of Zurich, Zürich, Switzerland; ^4^Clinical Psychology and Psychotherapy, University of Ulm, Ulm, Germany; ^5^Department of Clinical, Neuro, and Developmental Psychology, Vrije Universiteit Amsterdam, Amsterdam, Netherlands

**Keywords:** internet- and mobile-based intervention, internet use disorder, case report, social media use, student

## Abstract

**Background and aims:**

Internet use disorder (IUD), characterized as the inability to control one’s internet use, is associated with reduced quality of life and mental health comorbidities such as depression, substance abuse, or anxiety. Evidence-based treatment options are scarce due to the novelty of the diagnosis. Internet- and mobile-based interventions (IMI) may be an effective means to deliver psychological treatment to individuals with IUD as they address affected individuals in their online setting. This study presents a newly developed IMI for IUD disclosing treatment satisfaction and preliminary effects by exemplifying with a case report.

**Methods:**

The case of a female participant with IUD, characterized by an excessive use of social media, is analyzed. The case report follows the CARE guidelines and presents qualitative and quantitative outcomes regarding potential symptom reduction measured by the Internet Addiction Test (IAT) and Compulsive Internet Use Scale (CIUS), treatment satisfaction measured by the Client Satisfaction Questionnaire (CSQ) and feasibility by analyzing participant’s written feedback during treatment.

**Results:**

The case report shows that internet- and mobile-based interventions may be feasible in supporting an individual in reducing symptoms of IUD as well as depressive symptoms, anxiety and procrastination behavior. Treatment satisfaction was reported as good.

**Discussion and conclusions:**

This case report illustrates that IMIs can have the potential to be an easily accessible and possibly effective treatment option for IUD. Case studies on IMIs may provide insights into important mechanisms for symptom change. Further studies are needed to expand our understanding of this diverse disorder to provide adequate treatment.

**Clinical Trial Registration:**

https://clinicaltrials.gov/,DRKS00015314.

## Background

Internet use disorder (IUD) is characterized by excessive or poorly controlled preoccupations, urges, or behaviors regarding computer and internet use that lead to social or work-related impairment or distress (1). Pathological internet use can be divided into different subtypes related to both gaming and non-gaming internet activities. Non-gaming internet activities include problematic or pathological internet gambling, obsessive research and surfing, compulsive online shopping as well as excessive use of social networks and internet pornography (2–5). There is currently no standard definition of IUD in diagnostic manuals such as the Diagnostic and Statistical Manual of Mental Disorders [DSM-5; (6)] or the International Statistical Classification of Diseases and Related Health Problems [ICD-10; (7)]. However, in the updated version of the International Statistical Classification of Diseases and Related Health Problems [ICD-11, (7)], gaming and gambling disorder were incorporated as disorders due to addictive behaviors. Moreover, a section of other specified disorders related to addictive behaviors (6C5Y) was included to code further problematic addictive behaviors beyond gambling and gaming, e.g., social-network-use disorders, and pornography-use disorder. Diagnostic criteria provided in the ICD-11 for disorders due to addictive behaviors encompass functional impairment, loss of control over the problem behavior, neglect of social and work life, and excessive use of the internet despite negative consequences which may be episodic or recurrent [ICD-11; (7)]. In the DSM-5, internet-based gambling, as a part of IUD, is included in the Gambling Disorder diagnostic criteria (6) and Internet Gaming Disorder is defined as a “Condition for Further Study” (6). The worldwide IUD prevalence is currently estimated to be at around 7.0% (8). Women have shown to be especially at risk for excessive social network use (9). A Social Network Use Disorder in specific is discussed in recent research as a pathological use of social networks, which is more likely to occur among women and presents itself with similar comorbidities as IUD (10–12).

As comorbid symptoms, IUD may cause neurological complications, psychological distress, and social problems (13–15). In addition, high comorbidities with other mental disorders have been reported, especially affective and anxiety disorders, impulse control disorders, substance use disorders, and attention deficit hyperactivity disorder (1, 16–18). Impairment caused by IUD has also been found to include educational failure and reduced academic perspectives especially in teenagers and young adults (19, 20).

Evidence-based treatment for IUD is, however, scarce. The few randomized controlled trials existing have shown large effect sizes of cognitive-behavioral treatment (CBT) on IUD in terms of reducing time spent online and IUD symptoms (21–23). Still, in order to adequately approach the heterogeneity of IUD resulting in different impairments depending on preferred online activities, specialized and innovative treatment options have called to be further studied (2, 24–26). Previous case reports on treatment of IUD have focused on internet gaming (27, 28).

Treating IUD *via* the internet may appear contradictive at first, as it seems problematic in terms of additional time spent online. However, internet- and mobile-based interventions (IMIs) can contribute to practice controlled internet use, which is recommended as treatment goal instead of abstinence (29–31). IMIs also have the advantage to reach individuals through their common and attractive online setting who may otherwise not consult a therapist due to low treatment motivation and reduced readiness-to-change (32). Thus, IMIs can deliver specialized treatment with a low threshold and easy access for uptake (33, 34). The aim of this study was to give insights into the internet- and mobile-based treatment of IUD with a case report illustrating feasibility, symptom reduction and satisfaction at individual level. As studies on IMIs for IUD are lacking in Germany, the objective of the present work is to present the therapeutic manual of a newly developed IMI for IUD using a detailed case report of a patient who successfully completed the program and describe the course of treatment including treatment effects, potential barriers, and user satisfaction.

## Methods

### Treatment format

The intervention was CBT-based and consisted of the following six weekly core sessions: Goal setting and motivational interviewing (session 1), impulse control (session 2), problem solving (session 3), cognitive restructuring (session 4), self-worth (session 5), and relapse prevention (session 6). Four weeks after completion of the core sessions, a booster session was provided. The aim of the booster session was to support the user in reflecting and refreshing intervention content and strategies. The user could additionally choose between the following elective sessions: personal needs and values, sleep, relaxation, alcohol and affect regulation, appreciation and gratefulness, and procrastination (see Table 1). The core sessions took approximately 45–60 min to be completed. The user could continue with the following sessions once the previous session was completed. The intervention was guided self-help provided completely online on an internet-based platform of an eHealth provider. The intervention could be completed on any internet-ready device, i.e., PCs, laptops, smartphones, and tablets. The intervention included interactive elements (exercises, quizzes, testimonials, homework) and the user was able to answer questions *via* text boxes. The answers were then stored within the program and could be viewed by the eCoach and exported in a data format for qualitative evaluation. In addition to the core intervention content in the treatment sessions, the user could use Tiny Tasks, i.e., small exercises and motivational messages *via* smartphone three times a day. The aim of the tiny tasks was to help the user to transfer the intervention content into their daily lives. They consisted, e.g., of suggestions on how to implement the intervention strategies: “What has influenced your internet usage today?” or “Which features play a key role in contributing to the amount of time you spend online? Are you able to influence those features?”

**Table 1:** Overview of content of main sessions and elective sessions of the training.

Intervention content – Main sessions	Session
Goal setting and motivational interviewing	1
Impulse control	2
Problem solving	3
Cognitive restructuring	4
Strengthening self-worth	5
Relapse prevention	6
Booster session	7
**Intervention content – Elective sessions**	
Sleep	
Alcohol and affect regulation	
Appreciation and gratefulness	
Personal needs and values	
Procrastination	
Relaxation	

After completion of each session, the user received content-focused guidance by an eCoach who provided individually manualized feedback (35). The qualification of the eCoaches was at least a bachelor’s degree in psychology. The supervised eCoaches used a treatment manual with standardized text blocks which were individually adapted based on the input and overall progress of each user. Further, there was an internal messaging function on the intervention platform through which the user could contact the eCoach. For a detailed description of the IMI see the study protocol (36).

### Design of the case report

A case study was conducted as part of a larger RCT to evaluate the efficacy of the intervention. The case study was conducted to analyze and illustrate an individual course of treatment within the framework of the internet-based program. The case report follows the Case Reporting (CARE) guidelines (37). Exemplary symptom reduction and satisfaction of a female student with successful treatment outcome was described. Selection of treatment case considered representativeness of participant characteristics. Case selection followed criteria such as complete treatment course and the transferability of the case through the affiliation to a risk group as a student, as well as through the predominant use of social networks, which has shown to be a represented subtype of IUD among females (9, 11). As a student of young age, she is part of a target group that has been identified as a risk population for IUD (38).

After registering with a self-chosen email address on the study website, the participant received detailed information about the study procedure and was further informed about the possibility to withdraw from the intervention and/or study at any time without any negative consequences. The participant was asked to sign the informed consent together with a data security and confidentiality form. The participant gave informed consent for the participation in the RCT in general and the analysis of her single case in specific. To ensure pseudonymization, we used an individual participant ID number. On the intervention platform, the participant also registered also registered with a self-chosen anonymous email address. Treatment fidelity was assured as all participants received the same intervention on the online platform ensuring that the intervention has been consistently administered. Randomization and allocation of study participants was performed by an independent employee who was not otherwise involved in the study. All study participants were randomized in 1:1 ratio to the intervention or waitlist-control group. A research assistant not otherwise involved in the study performed block randomization with varying block sizes using an automated computer-based random integer generator (Randlist; Datinf GmbH, Tübingen, Germany). All procedures were consistent with the generally accepted standards of ethical practice approved by the Friedrich-Alexander Universität of Erlangen-Nürnberg ethics committee (54_18 B). The trial is registered in the German Clinical Trials Register (DRKS00015314).

To assess feasibility of the IMI, the participant’s course of treatment and the number of completed sessions are reported. We qualitatively evaluated the participant’s written content in each session and analyzed quantitative data from the online pre- (T1) and post-treatment assessments (T2) 7 weeks after randomization. Self-reported data was collected using a secure online-based assessment system (UNIPARK, 256-bit encrypted, EFS Survey, 2016).

The Internet Addiction Test [IAT; (39)] and the Compulsive Internet Use Scale [CIUS; (40)] were used to assess IUD symptoms (see Table 2). Other secondary outcomes included depression [PHQ-9; (41)], insomnia severity [ISI; (42)], anxiety [GAD-7; (43)], procrastination [GSP-K; (44)], alcohol abuse [AUDIT-C; (45)], worries [PSWQ-3; (46)], work-related impairment [WLQ; (47)], health related quality of life [AQoL-8D; (48)], and psychological wellbeing [WHO-5; (49)] (for a complete overview, see Table 3). Efficacy is indicated by reporting change scores. To analyze treatment satisfaction, the Client Satisfaction Questionnaire for internet interventions [CSQ; (50)] was used as well as qualitative analysis of the written feedback given by the participant after each session. The written content of the participant was assessed by open format answers to specific question on the intervention platform. The feedback was recorded on the platform and analyzed by an inductive approach. The feedback asked about (1) perceived usefulness of the session, (2) completion time, (3) treatment elements that the user liked, (4) exercises that were perceived as helpful, (5) components that were not perceived as helpful, (6) perceived support from the testimonials, and (7) suggestions for improvement.

**Table 2:** Overview of subscale values and total scores of IAT and CIUS at T1 and T2.

Questionnaire	Subscale	Items	T1	T2
IAT (39). Cut-off ≥49. Score range: 20–100 (higher scores reflect higher IUD)	Salience	5	10	8
	Neglect social life	2	7	4
	Neglect work	3	10	5
	Anticipation of internet use	2	8	6
	Excessive use	5	13	9
	Loss of control	3	12	8
	Total scores	20	65	44
CIUS (40). Cut-off ≥28. Score range: 14–56 (higher scores reflect higher compulsive internet use behavior)	Salience	3	7	6
	Withdrawal symptoms	1	3	2
	Coping	2	8	6
	Conflict	4	16	8
	Loss of control	4	15	10
	Total scores	14	49	32

**Table 3:** Overview of sum scores of measured comorbidities at T1 and T2.

Construct	Questionnaire	Sum scores	
		T1	T2
Depression	The Patient Health Questionnaire [PHQ-9; (41)]. Score range: 0–27 (higher scores reflect higher depressive symptoms)	17	14
Sleep	The Insomnia Severity Index [ISI; (42)]. Score range: 0–28 (higher scores reflect higher insomnia symptoms)	16	19
Anxiety	The Generalized Anxiety Disorder Scale [GAD-7; (43)]. Score range: 0–21 (higher scores reflect higher anxiety)	14	12
Procrastination	The General Procrastination Scale – short version [GSP-K; (44)]. Score range: 0–36 (higher scores reflect higher procrastination behavior)	31	23
Alcohol consumption	The alcohol use disorders identification test [AUDIT-C; (45)]. Score range: 0–12 (higher scores reflect higher alcohol consumption)	3	3
Worries	The Penn State Worry Questionnaire-Ultra Brief Version [PSWQ-3; (46)]. Score range: 0–18 (higher scores reflect higher worrying)	11	12
Work limitations	Work Limitations Questionnaire [WLQ; (47)]. Score range: 5–50 (higher scores reflect higher work limitations)	30	43
Quality of life	The Assessment of Quality of Life Instruments [AQoL-8D; (48)]. Score range: 35–175 (higher scores reflect lower quality of life)	76	81
Wellbeing	The WHO-Wellbeing Index [WHO-5; (49)]. Score range: 0–25 (higher scores reflect higher wellbeing)	18	15

## Case study

### Case history

Emma (pseudonym) is a 21-year-old female university student living with her parents and working part-time as a production assistance. She reported to be in a relationship and to have no financial issues. Emma is a first-time treatment seeker with no prior experience with internet-based health programs. She signed up for the intervention because of a constant impairing urge to stay “up to date” on the internet, i.e., refreshing the feeds of her preferred websites. This urge to be on the internet has caused severe difficulties for her to master her daily social and professional tasks. She indicated that at work she spent up to 5–6 h a day on the Internet. The online behavior in her leisure time is primarily characterized by the extensive use of social networks (e.g., Instagram, WhatsApp) and shopping portals in addition to setting up appointments and exchanging email messages. Emma emphasized using the internet as a distraction in stressful situations, which above all leads to problems in her relationship. She also reported efficiency problems and reduced mental well-being due to her excessive internet use. She became aware of the training on the homepage of her university and wanted to participate because the training met her need to cope with her problems on her own.

### Diagnostics

Emma met the criteria for IUD with a total score of 65 (cut-off ≥49) on the IAT (39) showing high pathological internet use with pronounced symptoms and a score of 49 (cut-off ≥28) on the CIUS (51), indicating problematic compulsive internet use behavior. Her online behavior was characterized by the extensive use of social networks and shopping portals in addition to exchanging email messages corresponding to the subtypes “social networks” and “obsessive research and surfing.” Comorbid symptoms of the participant comprised moderately severe symptoms of depression [17 on PHQ-9; (41)], a moderate level of anxiety [14 on GAD-7; (43)], a tendency to procrastination [31 on GPS-K; (44)], and moderate sleep problems [16 on ISI; (42)]. There were no symptoms of an alcohol use disorder [3 on AUDIT-C; (45)].

### Description of the treatment

Emma aimed at reduced, conscious, and deliberate smartphone use as her training goal. In session 1, she came to the conclusion that her high internet usage is maintained by the advantages that the internet dispels boredom, is fun, gives her a sense of belonging, and distracts her from problems. Her motivation for treatment resulted from relationship issues, the impairment with sleep and efficiency, as well as back pain and headaches (see Table 4).

**Table 4:** Emma’s identified personal advantages and disadvantages of her internet use.

Advantages of internet use	Disadvantages of internet use
+ The internet dispels boredom + The internet is fun + A perceived sense of belonging + A distraction from problems	Poor sleep Concentration issues Headaches Back pain Reduced efficiency Forgetfulness

Emma explained that she has realized through psychoeducation that her use of social networks significantly influences her self-esteem (session 3). She stated that she suffers from the perceived pressure to be perfect through social media. She indicated that online advertising gives her an embellished image of women that makes her feel inferior. She tries to alleviate these feelings of insufficiency by uploading edited images of herself on Instagram in order to receive positive feedback by her followers. To strengthen her self-esteem in real life (session 5), she planned mood-promoting self-care activities (e. g. taking a walk) and formulated affirmative statements about herself and her abilities (e.g., “I am open-minded and honest.”; “I am good at cooking and baking.”) during her IMI participation.

As a strategy to overcome strong urges to update her Instagram feed in the morning, she decided on one of the presented strategies in Session 2 and chose to distract herself by reading the newspaper. Planning positive activities with a weekly schedule to specify time and activity, such as doing yoga, reading a book, taking a bath, and scheduling targeted rewards (e.g., listening to music) served as alternatives to her internet use and strengthened her self-control.

In session 4 on cognitive restructuring, Emma identified the underlying vicious circle of her overall tendency to use the internet as an emotion regulation strategy and described these situations in which she tries to influence her feelings through the internet as “escape moments.” To regulate her feelings independently of the internet, Emma found the thought record for cognitive restructuring particularly helpful (see Table 5). She reported that the thought record enabled her in unpleasant situations (e.g., her boyfriend gazes after another woman), to identify negative thoughts (“She has prettier eyes than me”) and associated emotions such as feelings of worthlessness and self-doubt. By following the instructions of the thought record, she was able to develop positive and helpful thoughts (“My boyfriend loves me. If I start thinking positively, he will do so as well.”), which led to more pleasant feelings and a sense of stability and love in her partnership.

**Table 5:** Emma’s thought record of the instructed cognitive restructuring exercise.

Situation	Emotion/Feeling	Negative automatic thoughts	Alternative thoughts	Emotion/Feeling
“My boyfriend gazes after another woman. I distract myself with Instagram and followers complimenting me.”	Worthlessness, self-doubt	“She has prettier eyes than me, better lips and skin and a narrower nose.”	“My boyfriend loves me. If I start thinking positively, he will do so as well.”	More pleasant feelings, a sense of stability and love in the partnership.

In the following sessions, she repeatedly stated her satisfaction with the “new thoughts.” Following the “writing a letter to yourself”-exercise, Emma realized, that instead of attempting to distract herself with Instagram and followers complimenting her, she tried to become aware of negative thoughts and to take an appreciative attitude towards herself and her relationship (session 6): “Appreciation is very important and I’m getting it back bit by bit.”

To improve her efficiency problems, she chose the elective session on procrastination and tried out different strategies promoting effective time management. She also informed herself about sleep hygiene in another elective session and received strategies on healthy sleep, e. g. not exposing herself to screen light before going to bed. In the sessions “Appreciation & gratefulness” and “Personal needs & values,” she identified important values for herself (“I would like to be there for my family more often.”).

### Outcome

Regarding feasibility, Emma completed all seven core sessions, four elective sessions and four diary entries. On average, she required 0.5–1 h to complete one session and an average of 3 days to go through one core session (range 2–7 days). The participant opted to receive smartphone notifications to accompany the first two sessions.

With regard to symptom reduction, her self-reported symptoms of IUD had decreased from 65 to 44 at post-treatment (7 weeks after randomization) on the IAT corresponding to an improvement to a non-pathological level (cut-off ≥49). There was a decrease from 49 to 32 on the CIUS, showing a reduced symptom severity which was however still above the threshold (cut-off ≥28) for indicating a compulsive internet use (see Table 2). Reduced loss of control (T1: 12, T2: 8) and excessive usage (T1: 13, T2: 9) together with a decrease in perceived salience of internet-related stimuli (T1: 10, T2: 8) were observed. The participant also reported less conflicts in her social (T1: 7, T2: 4) and work environment (T1: 10, T2: 5). Within the qualitative written statements, Emma described a reduction of internet-related thoughts and reported to spend on average 4 h daily online, 3 h less than at the beginning of the training. The self-estimated desire to use the internet had decreased between the first and last week of training from 60–70 to 0%. With regard to comorbidities, the evaluation resulted in a slight reduction of depressive symptoms (T1: 17, T2: 14), anxiety (T1: 14, T2: 12), and procrastination behavior (T1: 31, T2: 23). There was a slight decrease in insomnia (T1: 16, T2: 19) symptoms (for an overview, see Table 3). Each of the main sessions was rated as helpful and the support by testimonials in the sessions was used continuously.

Emma rated the quality of the training as good (CSQ: total score = 21; range 8–32). She stated that she received the kind of treatment she wanted, and the intervention met most of her needs. She was largely satisfied with the level of provided support and the treatment helped her to deal more appropriately with her problems. She would consider the uptake of other online interventions if she needed help in the future and would recommend the intervention to a friend in need.

## Discussion

The aim of this case report was to illustrate feasibility, exemplary symptom reduction, and satisfaction of an IMI to treat IUD at individual level. The case study showed that the IMI can successfully support an individual in reducing symptoms of IUD and in achieving self-imposed treatment goals such as improved control over the internet use. Treatment satisfaction was reported as good. In addition to a reported decrease of time spent online and internet-related thoughts, the quantitative data showed a decline of symptoms of IUD, such as feeling less negligent towards work and social life. Furthermore, procrastination behavior tendencies have decreased, as well as the frequency and intensity of depressive symptoms and anxiety symptoms.

In this case report, cognitive restructuring to deal with negative thoughts and emotions and the impulse control strategies were assessed as particularly helpful to control the internet use in everyday life. By showing that IUD is related to many other mental health issues, such as procrastination or depression, it seems important to also address general mental health difficulties to enable comprehensive treatment. The flexibility of the intervention also allowed the participant to complete the intervention in her own pace. The detailed examination of the treatment course provides insight into influencing factors in the emergence and maintenance of IUD. The use of social networks as a coping strategy to regulate emotions in the short term mainly seems to contribute to the maintenance of the disorder but resulting in feelings of self-worthlessness in the long term that cause suffering. By establishing new emotion regulation strategies, such as developing positive and helpful new thoughts, this maintaining factor could be successfully addressed.

According to the merging of behavioral addictions with substance related disorders in the ICD-11 approach under the top level block “Disorders due to substance use or addictive behaviors” [ICD-11; (7)], IUD can be recently conceptualized as an addictive disorder. Previously, IUD was coded as an impulse control disorder in the ICD-10 [ICD-10; (7)]. Yet, there is also critique on the conceptualization towards disorders due to substance use, stating insufficient empirical evidence for IUD as an addictive disorder (52–58). Meta-analytic results show that unpleasant feelings being offline cannot be regarded as equivalent to the state of withdrawal from psychoactive substances (59). However, there is also evidence depicting similarities in brain activation (60) as well as underlying learning processes (61) for IUD and disorders due to substance use. Further, the complexity of IUD impedes the definition of a single diagnostic term. There are strong differences in the symptom patterns depending on the use of different online activities and subtypes, e.g., gaming, social network use, and pornography use (4, 62, 63). Accordingly, one generic diagnosis might be too inaccurate for the heterogeneity of problem behaviors in IUD and the selection of adequate treatment strategies (58). In future research, it might be essential to elaborate on the actual problem core rather than focusing on generic diagnoses.

The first limitation of this case report is the selection of a participant with a successful treatment course. Despite this selection bias, Emma’s case can be seen as representative due to her online activities and demographic characteristics. To generalize results, RCT data is needed. Second, the participant’s self-stated initial motivation to behavior change was rather high. Low motivation for change and ambivalence about internet use represents a common barrier to seeking treatment. Motivational issues should therefore be considered and addressed in the treatment of IUD. Third, complementary diagnostic instruments representing current classification approaches should be used in future studies to assess IUD. Fourth, only self-reported data was used, thus an influence of social desirability cannot be excluded. Fifth, the case report depicts an individual treatment course with individually chosen treatment components and exercises. Future studies should evaluate the use of individual selection of treatment components in a tailored compared to a standard approach. Treating IUD using a digital health intervention may be associated with patients continuing to spend time and possibly more time on the Internet in the short term as a result of participating in a digital intervention. However, an important goal of the intervention is to help patients gradually build up more activities in the offline setting so that, with the help of strengthened resources and alternative behaviors, they can reduce and control their Internet use in the long term. Moreover, limitations of IMIs in comparison to face-to-face treatments include potential risks in the therapeutic process, e. g. overlooking disease aspects, avoidance of difficult topics on the patient’s side, lack of nonverbal signals, or not being able to react appropriately to crises. However, there is evidence that IMIs show an utmost potential to reach burdened individuals which might otherwise not be reached by the health care system because of, i.e., the flexibility of IMIs in time and place and their low-threshold accessibility (32–34, 64).

## Conclusion

From the case report presented here, it can be concluded that an IMI might be a potentially feasible easy to access and effective treatment approach for IUD. If the available results can be confirmed in the randomized controlled efficacy study, IMIs could serve as a treatment option for people who prefer to achieve more control over their internet use.

## Data availability statement

The original contributions presented in the study are included in the article/supplementary material, further inquiries can be directed to the corresponding author.

## Ethics statement

The studies involving human participants were reviewed and approved by Friedrich-Alexander Universität of Erlangen-Nürnberg ethics committee (54_18 B). The participant gave written informed consent for study participation, data analysis and the case study’s publication.

## Author contributions

KB and DE designed the study. KB and EP analysed and interpreted the data. KB drafted the manuscript supervised by A-CZ. All authors participated in the review and revision of the manuscript and have approved the final manuscript to be published.

## Conflict of interest

DE reports to have received consultancy fees or served in the scientific advisory board of Minddistrict, Sanofi, Novartis, Lantern, Schoen Kliniken, and German health insurance companies (BARMER, Techniker Krankenkasse). DE and MB are stakeholders of the Institute for Health Trainings Online (HelloBetter), which aims to implement scientific findings related to digital health interventions into routine care. MB is stakeholder of mentalis a digital mental health company. HB reports to have received consultancy fees and fees for lectures or workshops from chambers of psychotherapists and training institutes for psychotherapists. A-CZ reports fees for lectures or workshops and for expert videos for an internet-based intervention.

The remaining authors declare that the research was conducted in the absence of any commercial or financial relationships that could be construed as a potential conflict of interest.

## Publisher’s note

All claims expressed in this article are solely those of the authors and do not necessarily represent those of their affiliated organizations, or those of the publisher, the editors and the reviewers. Any product that may be evaluated in this article, or claim that may be made by its manufacturer, is not guaranteed or endorsed by the publisher.

## Abbreviations

IUD: internet use disorder
SV: scale value
SV_pre,_ SV_post_: scale value pre- and post-treatment
T1: measurement point pre-treatment (baseline)
T2: measurement point post-treatment
IAT: internet addiction test
CIUS: compulsive internet use scale
IMI: internet- and mobile-based intervention
CBT: cognitive behavioral treatment
